# Supplementary material for: The interactome of intact mitochondria by cross-linking mass spectrometry provides evidence for coexisting respiratory supercomplexes
Source: Mol Cell Proteomics. 2017 Dec 8;17(2):216–32. doi: 10.1074/mcp.RA117.000470 (PMC5795388; doi:10.1074/mcp.RA117.000470)
Supplement: Supplemental Data [file supp_RA117.000470_133922_0_supp_23978_fzffwf.zip › spectra_annotation/new_mito_protein_spectra_annotation/39-1-486-1-663-1.pdf]

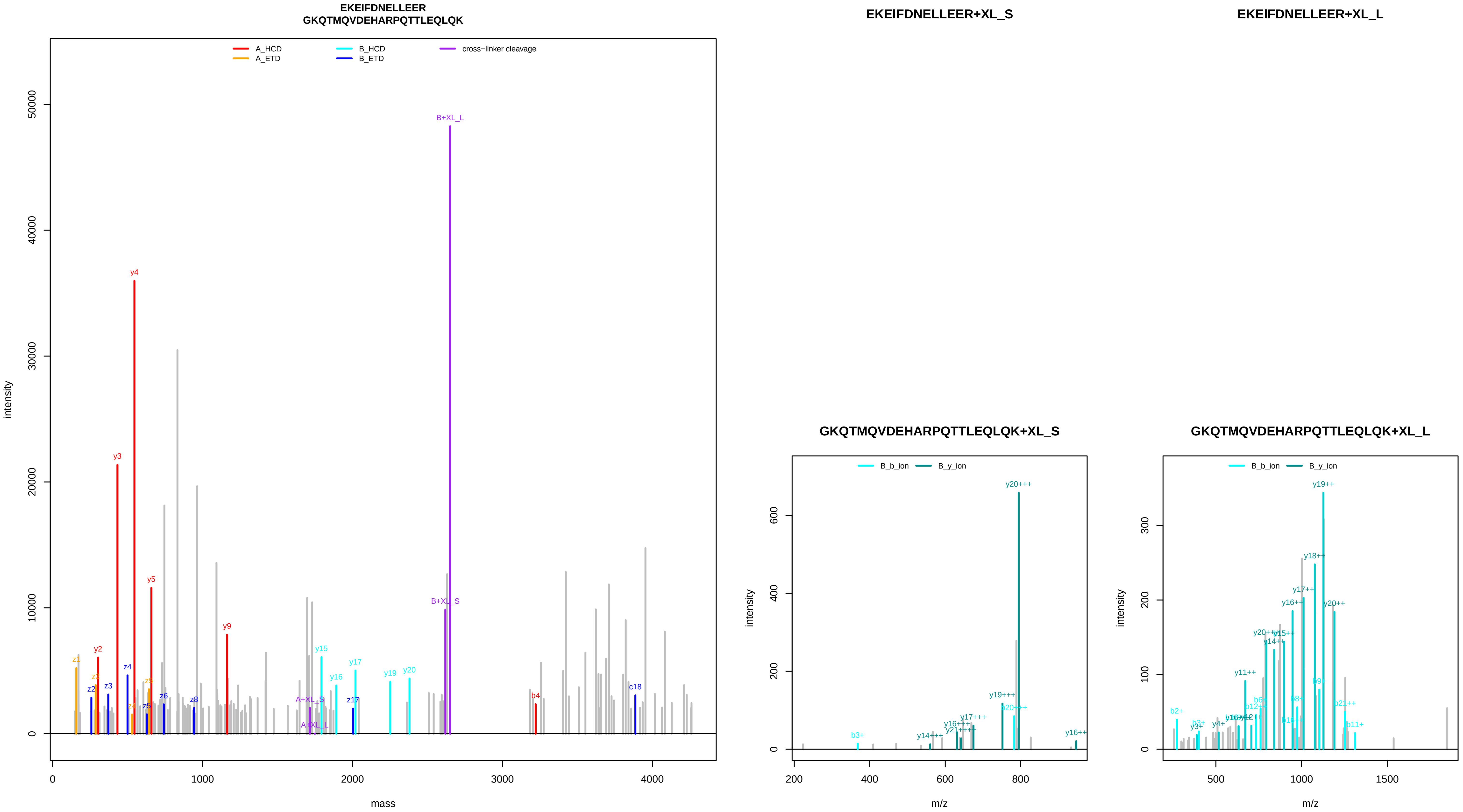
EKEIFDNELLEER
GKQTMQVDEHARPQTTLEQLQK
A_HCD
A_ETD
B_HCD
B_ETD
cross−linker cleavage
intensity
mass
B+XL_L
B+XL_S
A+XL_S
A+XL_L
y2
y3
y4
y5
y9
b4
z1
z2
z3
z4
z5
z6
z8
z17
c18
y15
y16
y17
y19
y20
EKEIFDNELLEER+XL_S
EKEIFDNELLEER+XL_L
GKQTMQVDEHARPQTTLEQLQK+XL_S
GKQTMQVDEHARPQTTLEQLQK+XL_L
B_b_ion
B_y_ion
m/z
y20+++
y19+++
y17+++
y16+++
y14+++
y21+++
b3+
y16++
y19++
y18++
y17++
y16++
y20++
y15++
y14++
y11++
b2+
b3+
y3+
y4+
b11+
